# Prevalence and determinants of minimum dietary diversity for women of reproductive age in Uganda

**DOI:** 10.1186/s40795-024-00858-6

**Published:** 2024-03-01

**Authors:** Derrick Kimuli, Florence Nakaggwa, Norah Namuwenge, Rebecca N. Nsubuga, Kenneth Kasule, Sheila Nyakwezi, Jimmy Odong, Paul Isabirye, Solome Sevume, Norbert Mubiru, Daniel Mwehire, Fatuma Matovu, Bonnie Wandera, Barbara Amuron, Daraus Bukenya

**Affiliations:** 1 Social & Scientific Systems, Inc., DLH Holdings company / United States Agency for International Development Strategic Information Technical Support Activity, Kampala, Uganda; 2 The United States Agency for International Development Uganda, US Mission Compound - South Wing, Kampala, Uganda

**Keywords:** Dietary diversity, Geographic location, Women, Education

## Abstract

**Background:**

Globally, over a billion women of reproductive age (WRA) suffer from some kind of undernutrition micronutrient deficiencies, and/or anemia as a result of inadequate dietary diversity. This leads to poor maternal and child health outcomes, however, there is limited research on population level research on minimum dietary diversity for women (MDD-W). This study assessed the prevalence and predictors of MDD-W among WRA in Uganda.

**Methods:**

This study was a secondary analysis of data from the lot quality assurance sampling (LQAS) survey conducted across 55 Ugandan districts between May and September 2022. Women of various ages were interviewed across 5 study subgroups that this study used to construct its study population (WRA). Descriptive analyses, tests for outcome differences, and multilevel mixed-effects logistic regression were conducted at a 5% statistical significance level using STATA version 17. The results were reported using Adjusted Odds Ratios (aOR) as the measure of the outcome.

**Results:**

The study analyzed responses from 29,802 WRA with a mean age of 27.8 (± 6.8) years. Only 8.8% (95% CI 8.5–9.3) achieved the MDD-W, the least proportion was observed in the South-Central region (3.13%). In the adjusted analysis, WRA who were older than 25 years (aOR 1.1, 95% CI 1.1–1.3, *p* < 0.001), had secondary education (aOR = 1.4, 95% CI 1.1–1.7, *p* = 0.003) or above (aOR = 1.7, 95% CI 1.3–2.2, *p* < 0.001), and used modern contraceptives (aOR = 1.1, 95% CI 1.0-1.3, *p* = 0.01) were more likely to achieve the MDD-W. Conversely, WRA who travelled longer distances to the nearest household water source (aOR = 0.8, 95% CI 0.7–0.9, *p* = 0.002) and those residing in larger households (aOR = 0.9, 95% CI 0.8-1.0, *p* = 0.019) were less likely to achieve the MDD-W.

**Conclusion:**

A low proportion of WRA met the MDD-W. Age, education level, household sizes and use of modern contraception were predictors of MDD-W among WRA in Uganda. MDD-W-related program efforts in Uganda should strengthen multisectoral collaboration with prioritization of younger women, education, household sizes and access to safe water sources.

## Background

In 2023, it was estimated that globally, two in three women of reproductive age (WRA) suffer from undernutrition, micronutrient deficiencies, and/or anemia [1]. This issue remains a public health concern, with progress being slow and undermined by various crises over the past few years, increasing the number of affected women [1–5]. The importance of adequate nutrition among WRA cannot be overstated. WRA are at a critical stage in their lives and may become mothers at some point, a physiologically demanding process that often increases the risk of maternal malnutrition [6]. This not only exacerbates poor maternal and child health outcomes but can also have a lasting impact on the health of their children, including an increased risk of childhood stunting [6–8]. In Uganda, national estimates showed that 32% of WRA are anemic and there are 336 maternal deaths per 100,000 live births [9]. Moreover, 10% of children had a low birth weight, and 29% of children under five years were stunted [9]. Adequate nutrition could help improve these outcomes by ensuring access to essential nutrients that are vital for maintaining good health and preventing nutritional deficiencies during pregnancy and lactation [10].

Minimum dietary diversity for women (MDD-W), *i.e. the consumption of at least five out of ten specified food groups*, is a proxy used to establish the adequacy of micronutrient intake among WRA at the population level [11]. This indicator considers the diversity of food groups consumed by women over a specified period, providing insights into their overall dietary quality, micronutrient intake and overall food security [11, 12]. Globally, one in three WRA meet the MDD-W criteria, indicating the need for further investigation and targeted interventions to improve women’s dietary diversity [1]. In Uganda, comprehensive population-level studies on MDD-W that can provide insights into regional and district findings are unavailable. Current studies are limited in scope to single districts [13–15]. The country’s population is predominantly rural, and many communities rely heavily on subsistence farming for their food supply [16]. This study aimed to determine the prevalence and predictors of MDD-W using data from a wider survey to present more representative findings that can influence nutrition programs in the country.

### Dataset used

The study utilized data from the 2022 lot quality assurance sampling (LQAS) survey carried out in specific Ugandan districts. LQAS is a widespread survey method that provides reliable estimates of service coverage at sub-district, district, and regional levels [17, 18]. This methodology is commonly used in public health surveys to quickly assess population-level conditions or processes, aiding in prompt decision-making. In Uganda, the survey process involved categorizing districts into “lots” based on predefined criteria such as population similarities and administrative factors. Within each lot, a representative sample of villages was selected using probability proportional to population size. At the village level, simple random sampling was employed to choose the initial household, acting as a reference point for the interview, utilizing a village household list if needed. Following the selection of the reference household, subsequent households were chosen, and eligible respondents were interviewed using simple random sampling. Only one respondent per household was interviewed. The LQAS surveys were district-led surveys conducted with support from the United States Agency for International Development. For more detailed information about the LQAS survey procedures in Uganda, interested individuals can refer to these references [18–22]. The 2022 LQAS survey was conducted from May to September, covering 55 districts in Uganda due to resource limitations. A total of 41,753 participants responded to the survey, the present study used questionnaires that were issued to WRA about their food consumption 24 hours preceding the survey.

### Study variables and measurements

The study’s dependent variable, MDD-W, is a dichotomous (yes or no) indicator of whether or not WRA consumed at least five out of ten defined food groups [1-Grains, white roots and tubers, and plantains, 2-Pulses (beans, peas and lentils), 3-Nuts and seeds, 4-Milk and milk products, 5-Meat, poultry and fish, 6-Eggs, 7-Dark green leafy vegetables 8-Other vitamin A-rich fruits and vegetables, 9-Other vegetables, and 10-Other fruits] the previous day or night. To construct the variable, the study utilised the 2021 updated recommendations from the Food and Agriculture Organization of the United Nations (FAO) [11]. The study identified independent variables by examining the underlying or enabling determinants of nutrition found within the LQAS survey. These determinants were aligned with the United Nations Children’s Fund (UNICEF) Conceptual Framework for Maternal and Child Nutrition [10]. The variables included were region, residence, education level, marital status, pregnancy, toilet facilities, distance to water source, health worker visit, household size and the use of modern contraception.

### Statistical analysis

The analysis was conducted in STATA version 17 [23]. In the bivariate analysis, the analysis used frequencies and percentages to summarize categorical data and means with standard deviation to summarize continuous data. The prevalence of MDD-W was summarized as percentage. The analysis assessed differences between MDD-W and the independent variables firstly using the Chi-squared test; *p*-values for each of the independent variables were presented in addition to the frequencies and proportions. In the multivariate analysis, multilevel mixed effects logistic regression analysis with clustering at the region and district levels was used. The analysis computed unadjusted odds ratios (uORs) and adjusted odds ratios (aORs) with corresponding 95% confidence intervals at a 5% statistical significance. Intraclass variability at both the region and district levels was also reported.

## Results

### Characteristics, the prevalence of MDD-W and bivariate analysis of differences in MDD-W receipt

The 2022 LQAS survey dataset contained responses from 41,753 participants. Of these, 29,802 (71.4%) were WRA with a mean age of 28.3 (± 6.8) years. The majority of the WRA were in the age group (15–25 years) (4,946, 50.3%), from the Lango region (5,397, 18.1%), rural areas (24,316, 81.6%), had primary school education (21,099, 70.8%), were married (24,206, 81.2%), were not pregnant (22,793, 86.5%), shared toilet facilities (16,061, 56.0%), lived less than 500 m from the nearest water source (20,535, 83.6%), had not received a health-related visit within the year preceding the survey (13,242, 74.2%), lived in a household with less than the mean household size (hhs) (18,399, 61.7%), and were not modern contraceptive users (21,586, 80.1%). Overall, only 8.8% (95% CI 8.5–9.2) of WRA achieved the recommended MDD-W. The results showed statistically significant differences (*p* < 0.001) in the achievement of the recommended MDD-W within regions, with more women in Bukedi achieving MDD-W compared to other regions. The results also showed that the proportion of women who achieved the recommended MDD-W was higher among urban women (10.5%), women with above secondary education (13.0%), pregnant women (10.0%), women who travelled a shorter distance to the nearest main water source (9.8%), women who had received a health worker visit (13.2%), women with a lower household size (9.3%), and women who used a modern contraceptive (10.2%). Table 1 shows the detailed findings on participant characteristics and the bivariate analysis results.

**Table 1:** Bivariate analysis of differences in MDD-W achievement

Variable	Categorization	Total (*N* = 29,802, %=100)	No (*n* = 27,173, %=91.18)	Yes *n* = 2629 (%=8.82)	P-value
Region	Acholi	5,176 (17.4)	4,761 (92.0)	415 (8.0)	< 0.001*
	Ankole	4,964 (16.7)	4,437 (89.4)	527 (10.6)	
	Bukedi	572 (1.9)	482 (84.27)	90 (15.7)	
	Bunyoro	1,429 (4.8)	1,235 (86.4)	194 (13.6)	
	Busoga	6,071 (20.4)	5,422 (89.3)	649 (10.7)	
	Kigezi	2,089 (7.0)	1,920 (91.9)	169 (8.1)	
	Lango	5,397 (18.1)	5,182 (96.0)	215 (4.0)	
	North Central	570 (1.9)	540 (94.7)	30 (5.3)	
	South Central	1,439 (4.8)	1,394 (96.9)	45 (3.1)	
	Tooro	2,095 (7.0)	1,800 (85.9)	295 (14.1)	
Residence	Urban	5,486 (18.4)	4,908 (89.5)	578 (10.5)	< 0.001*
	Rural	24,316 (81.6)	22,265 (91.6)	2,051 (8.4)	
Age category	15–25 years	14,946 (50.3)	13,730 (91.7)	1,216 (8.1)	< 0.001*
	26–35 years	9,747 (32.8)	8,804 (90.3)	943 (9.7)	
	36–49 years	5,039 (16.9)	4,572 (90.7)	467 (9.27)	
Level of education	None	1,846 (6.2)	1,690 (91.6)	156 (8.5)	< 0.001*
	Primary	21,099 (70.8)	19,438 (92.1)	1,661 (7.9)	
	Secondary	5,335 (17.9)	4,721 (88.5)	614 (11.5)	
	Above secondary	1,521 (5.1)	1,323 (87.0)	198 (13.0)	
Married	No	5,596 (18.8)	5,124 (91.6)	472 (8.4)	0.257
	Yes	24,206 (81.2)	22,049 (91.1)	2,157 (8.9)	
Currently pregnant (*n* = 26,351)	Yes	2,841 (10.8)	2,591 (91.2)	250 (8.8)	0.277
	No	22,793 (86.5)	20,801 (91.3)	1,992 (8.7)	
	Don’t know	717 (2.7)	642 (89.5)	75 (10.5)	
Pregnancy (*n* = 2,841)	Yes	2,068 (72.8)	1,861 (90.0)	207 (10.0)	< 0.001*
	No	773 (27.2)	730 (94.4)	43 (5.6)	
Shared toilet (*n* = 28,696)	Not shared	12,635 (44.0)	11,554 (91.4)	1,081 (8.6)	0.056
	Shared	16,061 (56.0)	14,583 (90.8)	1,478 (9.2)	
Distance to water source (*n* = 24,571)	less than 500 m	20,535 (83.6)	18,529 (90.2)	2,006 (9.8)	< 0.001*
	500 m or more	4,036 (16.4)	3,770 (93.4)	266 (6.6)	
Health worker visit (*n* = 17,837)	Yes	4,249 (23.8)	3,689 (86.8)	560 (13.2)	< 0.001*
	No	13,242 (74.2)	12,267 (92.6)	975 (7.4)	
	Don’t know	346 (2.0)	315 (91.0)	31 (9.0)	
Household size (hhs) (mean = 5.2 (± 2.4)	<=Mean	18,399 (61.7)	16,689 (90.7)	1,710 (9.3)	< 0.001*
	>Mean	11,403 (38.3)	10,484 (92.0)	919 (8.1)	
Modern contraception (*n* = 26,961)	No	21,586 (80.1)	19,748 (91.5)	1,838 (8.5)	< 0.001*
	Yes	5,375 (19.9)	4,834 (89.9)	541 (10.1)	

N = overall total, n = subtotal, *Denotes statistical significance at *p* < 0.01

### Prevalence of MDD-W by district and region

**Fig. 1:**
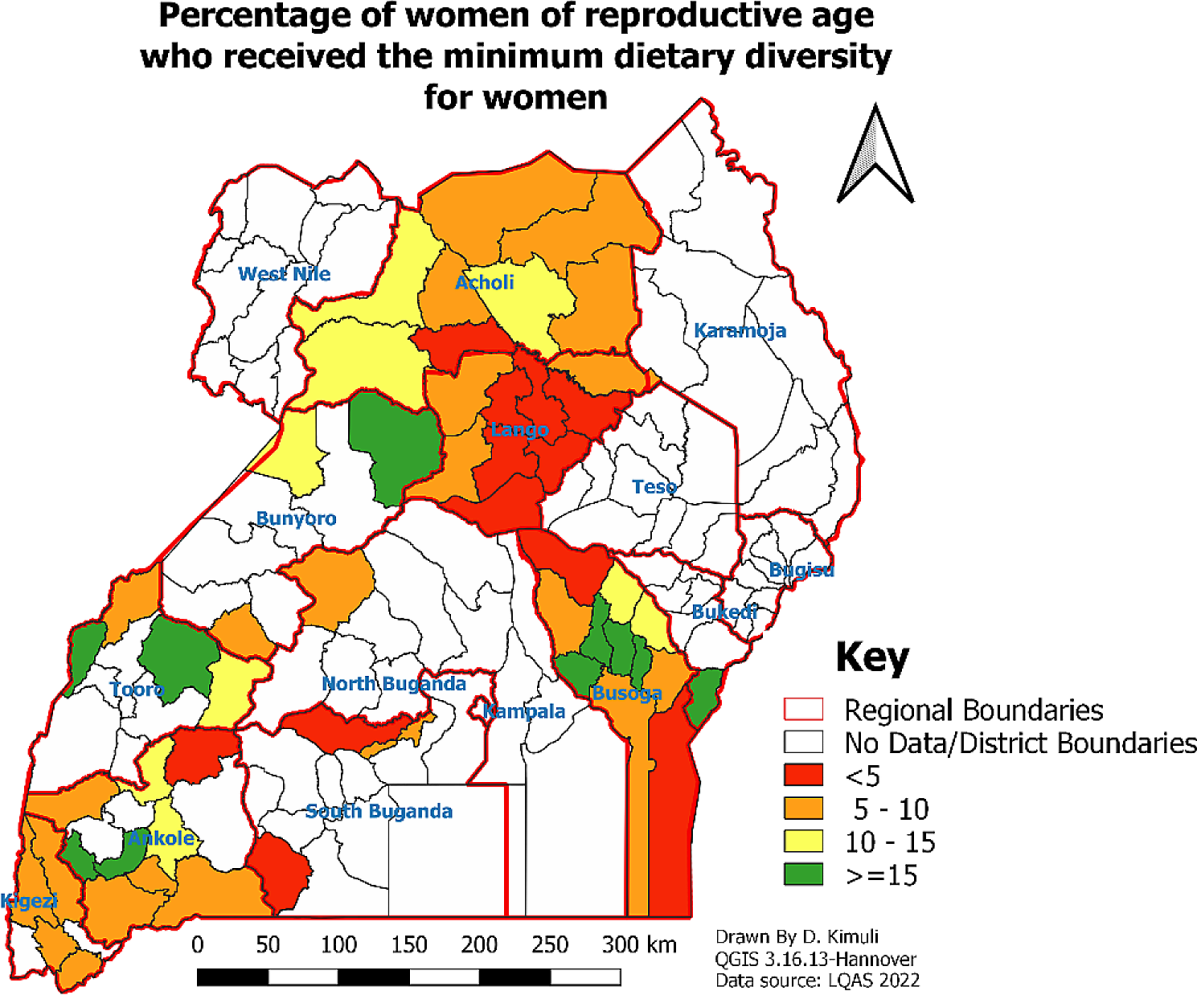
Achievement MDD-W among WRA by region and district

Overall, only 8.8% of women achieved the MDD-W. The lowestproportion of WRA who achieved an adequate MDD-W was observed in the South Central region (3.1%) and the Lango region (4.0%), while the highest proportion was observed in the Bukedi region (15.7%). Less than 1% of WRA in the districts of Lira, Rakai, Buyende, Omoro, Gomba, and Dokolo achieved an adequate MDD-W. More than 15% of WRA in the districts of Iganga, Mitoma, Luuka, Jinja, Busia, Bugweri, Kyenjojo, Sheema, Bundibujo, and Kiryandongo achieved an adequate MDD-W. Figure 1 shows the geographic differences in adequate MDD-W achievement by WRA.

### Factors associated with receiving the MDD-W among children WRA

Table 2 shows the multivariate analysis of factors associated with the achievement of MDD-W among WRA. The intraclass variability at the regional and district level was 6% and 18%, respectively. In the unadjusted analysis, WRA were more likely to achieve the MDD-W if they were over 25 years old (uOR = 1.1, 95% CI 1.1–1.3, *p* = 0.001) had secondary education (uOR = 1.4, 95% CI 1.1–1.6, *p* = 0.002) or above (aOR = 1.7, 95% CI 1.3–2.2, *p* < 0.001), and used a modern contraceptive method (uOR = 1.2, 95% CI 1.1–1.3, *p* < 0.001). They were less likely to achieve the MDD-W if they travelled a longer distance to the nearest water source (uOR = 0.8, 95% CI 0.7–0.9, *p* = 0.001), or lived in households with a higher number of persons (uOR = 0.9, 95% CI 0.8-1.0, *p* = 0.028). In the adjusted analysis, WRA who were older than 25 years (aOR 1.1, 95% CI 1.1–1.3, *p* < 0.001), had secondary education (aOR = 1.4, 95% CI 1.1–1.7, *p* = 0.003) or above (aOR = 1.7, 95% CI 1.3–2.2, *p* < 0.001), and who used modern contraceptives (aOR = 1.1, 95% CI 1.0-1.3, *p* = 0.01) were still more likely to achieve the MDD-W. However, WRA who travelled a longer distance to the nearest household water source (aOR = 0.8, 95% CI 0.7–0.9, *p* = 0.002) and those residing in households with sizes larger than the mean household size (aOR = 0.9, 95% CI 0.8-1.0, *p* = 0.019) were less likely to achieve the MDD-W.

**Table 2:** Factors associated with receiving MDD-W among children WRA

Variable	Unadjusted OR (95%CI)	P-value	ICC-Region	ICC-Region + District	Adjusted OR (95%CI)	P-value
**Residence**						
Urban	1 (Reference)		0.06	0.15	1 (Reference)	
Rural	0.97 (0.8–1.1)	0.581			1.1 (1.0-1.2)	0.131
**Age category**						
15–25 years	1 (Reference)		0.06	0.15	1 (Reference)	
26–35 years	1.1 (1.1–1.3)	0.001			1.2 (1.0-1.3)	0.005*
36–49 years	1.1 (1.0-1.3)	0.046			1.2 (1.1–1.4)	0.002*
**Education level**						
None	1 (Reference)		0.06	0.14	1 (Reference)	
Primary	1.0 (0.8–1.2)	0.935			1.0 (0.8–1.2)	0.810
Secondary	1.4 (1.1–1.6)	0.002			1.4 (1.1–1.8)	0.001*
Above secondary	1.6 (1.3–2.1)	< 0.001			1.7 (1.3–2.2)	< 0.001*
**Distance to water source**						
less than 500 m	1 (Reference)	0.001	0.06	0.14	1 (Reference)	0.002*
500 m or more	0.8 (0.7–0.9)				0.8 (0.7–0.9)	
**Household size**						
<=mean hhs	1 (Reference)	0.028	0.06	0.15	1 (Reference)	0.019*
>mean hhs	0.9 (0.8-1.0				0.9 (0.8-1.0)	
**Modern contraception**						
No	1 (Reference)	< 0.001	0.06	0.15	1 (Reference)	0.01*
Yes	1.2 (1.1–1.3)				1.1 (1.0-1.3)	

*Note* Exponentiated coefficients are for odds ratios; 95% confidence intervals in brackets; Findings from a multilevel logistic regression (clustering by Region). Intraclass Correlation Coefficient (ICC) Region = 0.06: ICC District nested in region = 0.18

* Denotes statistical significance at *p* < 0.05

## Discussion

The prevalence and factors influencing MDD-W among WRA in 55 districts that conducted the LQAS 2022 survey in Uganda were assessed in this study. The findings showed that only 8.8% of WRA achieved the MDD-W and highlighted significant differences in MDD-W among WRA across the country. The likelihood of MDD-W achievement was linked to higher education, the use of modern contraceptives and close access to a household water source.

Our findings, indicating an MDD-W prevalence rate of 8.8%, underscore potential challenges in accessing and/or consuming a diverse range of essential micronutrients by WRA during the reproductive years. Comparable findings were observed in the longitudinal Uganda National Panel Survey with MMD-W rates dropping from 14 to 11% between 2018/19 and 2019/20 [24]. The implications of the results extend beyond the immediate concern of a low MDD-W prevalence. Malnutrition among WRA can lead to adverse health outcomes, affecting not only maternal well-being but also influencing pregnancy outcomes and potentially contributing to an increased risk of childhood stunting [6, 8]. While the study participants predominantly resided in rural areas, over 70% of the Uganda population lives in such areas [25]. Previous studies have hinted at various factors contributing to suboptimal dietary diversity among WRA in Uganda including low income, reliance on rain-fed agriculture, and seasonally limited food availability and accessibility [13, 26]. Consequently, WRA may face financial constraints and limited market access, making it challenging for them to obtain a variety of nutrient-rich foods.

Our study echoes findings from Burkina Faso, emphasizing the geographical variation in achieving MDD-W [12]. While the South Central, Acholi and Lango regions had the lowest proportions of WRA receiving MDD-W, and Bukedi had the highest. Some districts reported alarmingly low MDD-W proportions, while others showed more promising results possibly influenced by diverse factors. Uganda’s tropical climate and varied geographic features, including mountains and lakes, affect farming patterns, contributing to regional differences. Some regions experience two rainy seasons and two dry seasons, however some parts of the country experience only one season of each [27–30]. Notably, the Bukedi region experiences two planting and harvesting seasons, unlike the Lango and Acholi regions which could influence the availability and consumption of diverse foods [13]. Sample limitations in the South Central region may explain the observed low MDD-W prevalence with only 2 out of 13 districts selected, the findings are inconclusive. On the other hand, ethnic traditions, influenced by Uganda’s multi-ethnic composition, also impact regional crop choices [31, 32]. Developmental indices, varying across regions, also influence nutritional habits and are underlying determinants of malnutrition [9, 10, 25]. Regardless of the factors at play, the regional differences in achieving adequate MDD-W in Uganda are concerning. Ongoing initiatives for district-led nutrition planning reflect of regional their regional differences that remain to be overcome [33, 34].

The study findings regarding an association between MDD-W with education, modern contraceptive use, household size and distance to water sources highlight underlying determinants of nutrition [10]. Women with higher levels of education are more likely to have access to information about nutrition and healthy eating [35, 36]. They are also more likely to have the skills and resources to prepare and cook a variety of healthy foods [37]. Women with access to modern contraceptives could be more likely to be able to plan their pregnancies, space their births and have smaller household sizes. Moreover, they could have also benefited from nutrition education at the health facility [38]. This gives them more time to contribute to or afford aspects that can lead to better dietary diversity. Water security and its influence on nutrition well-being are still a critical aspect that needs further evidence such as provided by Miller and others [39] upon which the present study builds. Women who have to travel long distances to access a water source have reduced time for meal preparation and cooking which may influence their decision-making [10].

One notable finding of the study was that younger women were less likely to achieve the MDD-W compared to older women. Studies in Uganda [15] and Ethiopia [40] have found similar results highlighting possible persistent undernutrition among younger women in Uganda. The observed difference may be attributed to several factors, such as the economic status and cooking experience of older women, potentially enabling them to access and prepare a more diverse range of foods [37]. Additionally, older women tend to possess better knowledge and skills in making healthy dietary choices, possibly due to having more exposure to nutrition education messages or having more time to learn about nutrition [36, 41]. It is essential to recognize the potential consequences of inadequate nutrition during this pivotal life stage for women. Insufficient dietary diversity can lead to various health challenges, including nutritional deficiencies and anemia, adversely affecting their overall health and well-being [42, 43]. Consequently, the findings underscore a systemic challenge in attaining MDD-W during adolescence and early adulthood for women in Uganda, warranting continued attention and targeted interventions.

This study stands out as one of the few that examines MDD-W using LQAS data in Uganda, a dataset not commonly utilized for such analyses. The research offers valuable insights into the current situation of MDD-W in the country, as LQAS data allows for reliable district, regional, and national estimates. Notably, the study’s strengths include a large sample size, ensuring representation from various districts and regions and enhancing its generalizability. However, the study has some limitations that should be considered when interpreting or using the findings. One drawback of the study is that it is based on data from the LQAS survey which relies on self-reported data, introducing the potential for bias as respondents may not accurately convey their experiences [44, 45]. Moreover, the LQAS 2022 survey’s coverage was limited to 55 districts in Uganda and MDD-W may be affected by the season of data collection, a factor the present study was unable to account for due to limited data [13]. Lastly, the study did not control for additional factors that may impact MDD-W, making it challenging to discern the sole influence of the analyzed factors [10]. Despite these limitations, this study provides an in-depth discussion by considering known knowledge to interpret the findings in their context and offer potential solutions that can serve as building blocks towards improving the MDD-W status of WRA in Uganda.

## Conclusion

The prevalence of MDD-W among WRA in Uganda is low with notable differences in various regions and districts. The study findings showed that age, education, access to modern contraceptives and clean water are crucial for promoting dietary diversity among WRA. To improve dietary diversity among WRA, program efforts need to contextualize region and district drivers and develop targeted plans. Moreover, programs focusing on women’s education and contraceptive use, which can indirectly improve household nutrition are necessary [10]. By helping communities understand how education and family planning positively impact their nutrition, we can encourage them to adopt these interventions. Leveraging existing platforms like antenatal care visits, schools, or community health programs can efficiently integrate women’s nutrition education with other essential services, reaching a broader audience with valuable information. Overall, the study findings emphasize the call for multisectoral efforts to improve nutrition among WRA. Addressing factors beyond food availability and access, and integrating education and family planning initiatives, can enhance dietary diversity. While integrating services may present challenges and strain resources [46, 47], the findings suggest that integrating family planning into various service delivery outlets and collaborating with other government sectors like education, water, and sanitation can lead to real improvements in dietary diversity among WRA [33, 48].

## Data Availability

The data that support the findings of this study are available upon reasonable request from Social & Scientific Systems, Inc., a DLH Holdings Company. In compliance with our institutional data-sharing policy, the investigators are unable to publicly provide the data as part of the manuscript submission. To obtain the data from Social & Scientific Systems, Inc., a DLH Holdings Company, interested researchers should contact Daraus Bukenya (Dr.) at Daraus.Bukenya@dlhcorp.com, Barbara Amuron (Dr.) at Barbara.Amuron@dlhcorp.com or the DLH Institutional Review Board at IRBHelp@dlhcorp.com. Alternatively, researchers may choose to request the data directly from the respective districts. If researchers prefer to request data from specific districts, they may reach out to the corresponding district offices through the Ministry of Local Government at ps@molg.go.ug. Requests for data from Social & Scientific Systems, Inc., a DLH Holdings Company will be subject to review by the IRB Review Team, ensuring adherence to ethical and legal considerations.
